# Prognostic Impact of Pattern of Mandibular Involvement in Gingivo-Buccal Complex Squamous Cell Carcinomas: Marrow and Mandibular Canal Staging System

**DOI:** 10.3389/fonc.2021.752018

**Published:** 2022-03-03

**Authors:** Abhishek Mahajan, Navnath Dhone, Richa Vaish, Ankita Singhania, Akshat Malik, Kumar Prabhash, Ankita Ahuja, Nilesh Sable, Pankaj Chaturvedi, Vanita Noronha, Sarbani Gosh Laskar, Ujjwal Agarwal, Shreya Shukla, Gouri Pantvaidya, Prathamesh Pai, Atanu Bhattacharjee, Vijay Patil, Asawari Patil, Munita Bal, Swapnil Rane, Shivakumar Thiagarajan, Anil D’ Cruz

**Affiliations:** ^1^ Department of Radiodiagnosis and Imaging, Tata Memorial Hospital, Homi Bhabha National Institute, Mumbai, India; ^2^ Senior Resident Department of Radiodiagnosis, Tata Memorial Hospital, Homi Bhabha National Institute, Mumbai, India; ^3^ Department of Head and Neck Surgery, Tata Memorial Hospital, Homi Bhabha National Institute, Mumbai, India; ^4^ Department of Medical Oncology, Tata Memorial Hospital, Homi Bhabha National Institute, Mumbai, India; ^5^ Department of Radiation Oncology, Tata Memorial Hospital, Homi Bhabha National Institute, Mumbai, India; ^6^ Section of Biostatistics Centre for Cancer Epidemiology, Tata Memorial Centre Homi Bhabha National Institute, Mumbai, India; ^7^ Department of Pathology, Tata Memorial Hospital, Homi Bhabha National Institute, Mumbai, India

**Keywords:** head and neck squamous cell carcinoma, oral cancer (OC), AJCC 8th edition, gingivo-buccal squamous cell carcinoma, imaging—computed tomography, imaging, prognostic model, outcome assessment

## Abstract

**Purpose:**

To study the pattern of mandibular involvement and its impact on oncologic outcomes in patients with gingivo-buccal complex squamous cell carcinoma (GBC-SCC) and propose a staging system based on the pattern of bone involvement (MMC: Marrow and mandibular canal staging system) and compare its performance with the 8th edition of the American Joint Committee on Cancer (AJCC8).

**Methods:**

This retrospective observational study included treatment-naïve GBC-SCC patients who underwent preoperative computed tomography (CT) imaging between January 1, 2012, and March 31, 2016, at a tertiary care cancer center. Patients with T4b disease with high infratemporal fossa involvement, maxillary erosion, and follow-up of less than a year were excluded. The chi-square or Fisher’s exact test was used for descriptive analysis. Kaplan–Meier estimate and log-rank test were performed for survival analysis. Multivariate analysis was done using Cox regression analysis after making adjustments for other prognostic factors. p-Value <0.05 was considered as significant. Based upon the survival analysis with different patterns of bone invasion, a new staging system was proposed “MMC: Marrow and mandibular canal staging system”. “Akaike information criterion” (AIC) was used to study the relative fitted model of the various staging (TNM staging—AJCC8) with respect to survival parameters.

**Results:**

A total of 1,200 patients were screened; 303 patients were included in the study. On radiology review, mandibular bone was involved in 62% of patients. The pattern of bone involvement was as follows: deep cortical bone erosion (DCBE) in 23%, marrow in 34%, and marrow with the mandibular canal in 43% of patients. Patients with DCBE and no bone involvement (including superficial cortical) had similar survival [disease-free survival (DFS) and locoregional recurrence-free survival (LRRFS)], and this was significantly better than those with marrow with or without mandibular canal involvement (for both DFS and LRRFS). Patients with DCBE were staged using the MMC, and when compared with the AJCC8, the MMC system was better for the prediction of survival outcomes, as AIC values were lower compared with those of the AJCC8. There was a significant association (p = 0.013) between the type of bone involvement and the pattern of recurrence.

**Conclusions:**

For GBC-SCC, only marrow with or without mandibular canal involvement is associated with poorer survival outcomes. As compared with the AJCC8, the proposed Mahajan et al. MMC staging system downstages DCBE correlates better with survival outcomes.

## Introduction

Squamous cell carcinoma is the most common histology of the oral cavity cancers. There are a multitude of factors that impact the prognosis of patients with these tumors. Amongst these, mandibular bone erosion (through the cortical bone of the mandible: deep cortical and/or marrow) is found to be an important factor (1–5). According to widely accepted staging systems, its presence is considered to be stage T4a (6). The probability of mandibular bone erosion is higher with buccal mucosa lesions in close proximity to the mandible and gingival cancers, which occur due to invasion of the mandible through the occlusal surface (7–9).

Over recent years, it has often been argued that mandibular bone erosion needs to be characterized further. The Japan Society for Oral Tumors (JSOT) has defined T4 cancer as the invasion of the mandibular canal (10–12). Ebrahimi et al. based the T stage on size and depth of invasion for tumor categories T1–T3 and T4 in the presence of marrow invasion (13). In contrast, a few reports have suggested that tumor size correlates with adverse prognosis and that bone invasion is not an independent predictor of survival (14–16). On the contrary, some studies have reported that tumor size and marrow invasion are independent predictors of reduced survival (13, 17–19). In view of such varied evidence and lack of clarity, this study aims to evaluate the association of various patterns of mandibular bone involvement and their impact on survival. Based upon the findings, we also endeavored to develop a staging system that would reflect the implications of various types of bone invasion-superficial cortical erosion (erosive bony involvement), deep cortical erosion (infiltrative bony involvement), marrow involvement (infiltrative bony involvement), and mandibular canal involvement (infiltrative bony involvement), as assessed on imaging in a better way.

## Material and Methods

This is a retrospective study on treatment-naïve gingivo-buccal complex squamous cell carcinoma (GBC-SCC) patients who underwent preoperative CT imaging between January 1, 2012, and March 31, 2016, at a tertiary care cancer center. The patients who underwent treatment with curative intent were included. Since surgery is the mainstay of treatment for these cancers, only those patients who underwent definitive surgical management at our center were included in the study.

### Patients

**Figure 1:**
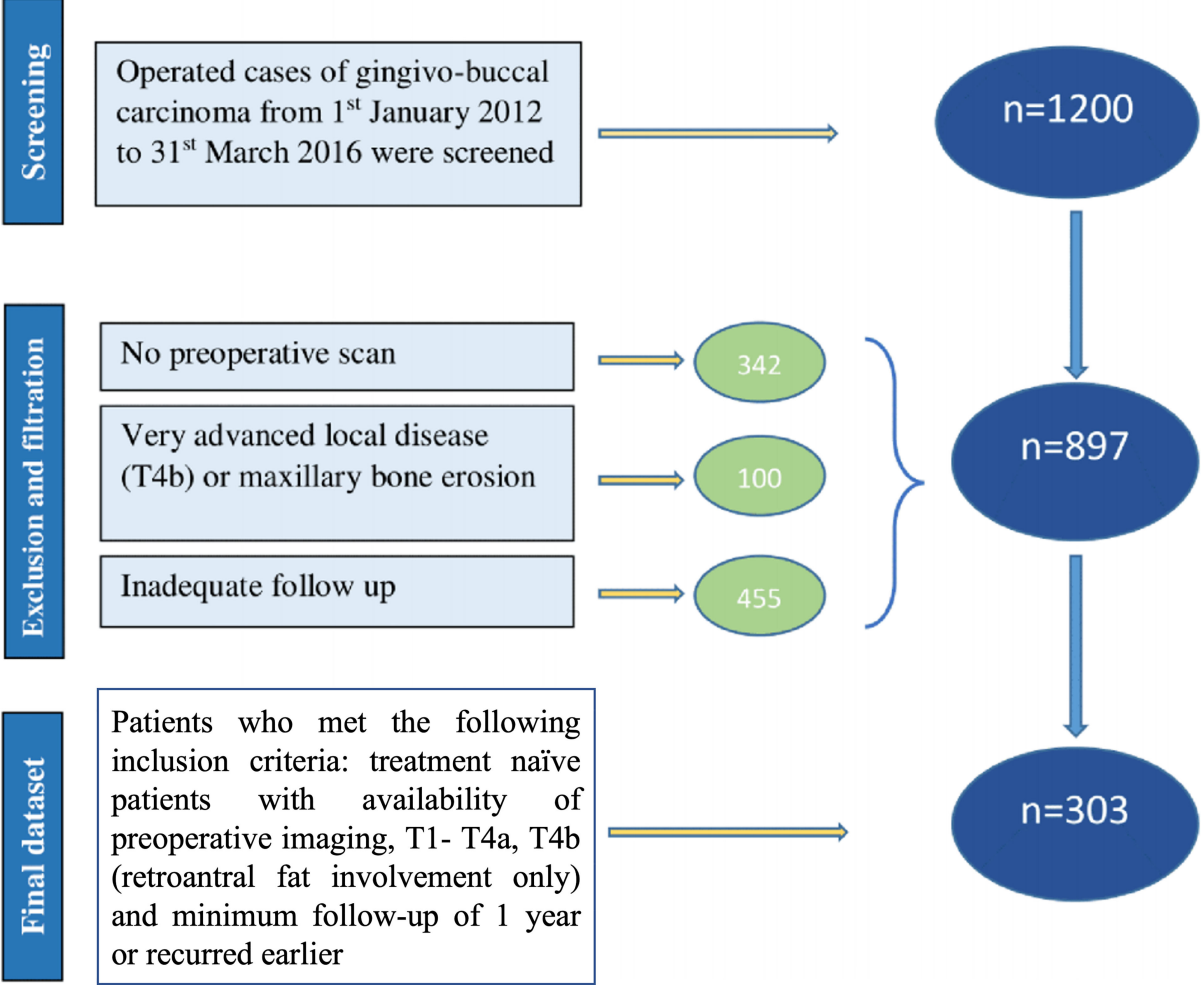
Consort diagram; 1,200 patients were screened, and 303 were included who met the inclusion criteria.

Overall, 1,200 patients were screened. We excluded patients with stage T4b with high infratemporal fossa involvement, maxillary erosion, those with follow-up of less than 1 year, and cases where digital imaging and communications in medicine (DICOM) images were not available for review. Analysis was performed on 303 patients in our study (**Figure 1**). The Institutional Ethics Committee approval was obtained. Since it is a retrospective study, the waiver of consent was granted. The demographic, treatment, histopathological, and follow-up details were obtained from the electronic medical records.

### Image Evaluation

Two senior head and neck radiologists with experience of over 10 and 6 years and one junior radiologist with experience of over 3 years reviewed the CT images independently (AbM, NS, and ND, respectively). The imaging review was performed on reconstructed DICOM data. The soft-tissue algorithm and bone window or bone algorithm reformations and axial images were analyzed on a volume viewer integrated within the picture archiving and communication system (PACS) using triangulation.

**Figure 2:**
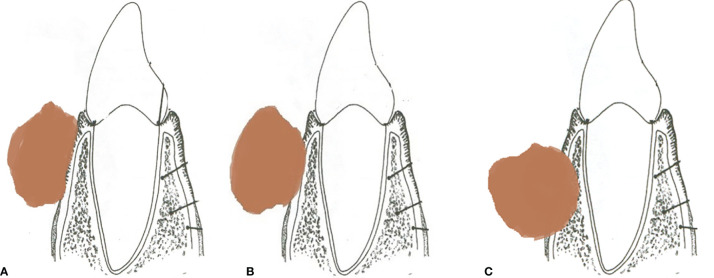
**(A)** Line diagram of tumor eroding the superficial cortex. **(B)** Tumor with deep cortical erosion. **(C)** Tumor with marrow involvement.

The various patterns of bone involvement reported on imaging were as follows: erosive infiltration, i.e., superficial cortical erosion with subtle outer cortical erosion without complete breach. Infiltrative invasion included deep cortical erosion with outer cortical breach and disease reaching up to the inner cortical layer, marrow involvement with disease eroding both the cortices and reaching up to the mandibular marrow, and mandibular canal involvement with disease eroding the inferior alveolar canal, obliteration of fat, or excessive enhancement within the mandibular foramen, with or without widening or erosion of the foramen, which was regarded as the perineural spread. **Figure 2** shows a line diagram of the described patterns of mandibular involvement. As the 8th edition of the American Joint Committee on Cancer (AJCC8) does not consider superficial cortical erosion for upstaging the disease, patients with superficial cortical erosion were included with patients having no bone erosion.

### Pathology Evaluation

The pathology reports of all tumors exhibiting bone invasion on imaging were reviewed. The bone invasion was categorized as present or absent in the final report. In cases where there was inadequate information regarding the extent of bone invasion, the second review of the pathology slides was performed by a senior head and neck pathologist (SR, AP, and MB).

### Statistical Considerations

The analysis was performed using SPSS version 21 and R software (IBM Corp). The chi-square or Fisher’s exact test was used for descriptive analysis. The overall survival (OS) was calculated from the date of surgery to death due to any cause. Disease-free survival (DFS) was defined from the date of surgery to any disease recurrence. Locoregional recurrence-free survival (LRRFS) was calculated from the date of surgery to the locoregional recurrence. The patients were censored if they were lost to follow-up or on the last follow-up date in case the event did not occur. Kaplan–Meier estimate and log-rank test were performed for survival analysis. Multivariate analysis was done using Cox regression analysis after making adjustments for other prognostic factors. p-Value <0.05 was considered significant.

#### MMC: Marrow and Mandibular Canal Staging System

Based upon the survival analysis with different patterns of bone invasion, a new staging system was proposed, “*MMC: Marrow and mandibular canal staging system*” (**Table 1**). The patients with no bone erosion/superficial cortical erosion and deep cortical bone erosion were staged based on the size and depth of invasion. Only marrow invasion with or without mandibular canal involvement was considered to be T4a. The patients were restaged according to this system, and this staging system was compared with the AJCC8 staging system.

**Table 1:** Marrow mandibular canal staging system.

T stage	Definition
**T1**	Tumor ≤ 2 cm in greatest dimensions with DOI ≤ 5 mm
**T2**	Tumor ≤ 2 cm in greatest dimensions with DOI >5 mm and ≤10 mm Tumor >2 to ≤4 cm in greatest dimensions with DOI ≤ 10 mm
**T3**	Tumor >2 to ≤ 4 cm in greatest dimensions with DOI > 10 mm Tumor > 4 cm in greatest dimensions with DOI ≤ 10 mm
**T4a**	Tumor > 4 cm in greatest dimensions with DOI >10 mm Tumor invades into mandibular marrow* with or without mandibular canal, maxillary sinus, retroantral fat or skin of face.
**T4b**	Tumor invades masticator space, pterygoid plates, or skull base or encases internal carotid artery

DOI, depth of invasion.

*Deep cortical erosion is not considered to be T4a in the marrow mandibular canal (MMC) staging system.

“Akaike information criterion” (AIC) was used to study the relative fitted model of the various staging (TNM staging—AJCC8) with respect to OS, DFS, and LRRFS. AIC estimates the best-fitted model, relative to other models, thus providing the means for each model selection. R software and survival package were used to calculate the AIC values.

## Results

### Patient Characteristics

We screened 1,200 patients, out of whom 303 patients met the inclusion criteria and were included in the final analysis. The mean age of the cohort was 52.86 years (30 to 84 years). Of these, 258 (85%) were males and 45(15%) were females. The personal habits revealed that most of the patients 263 (86.8%) were tobacco chewers/smokers; 27 (8.9%) had multiple habits. Out of 303 patients, 261 (86%) underwent segmental mandibulectomy, and 42 (14%) underwent marginal mandibulectomy. A total of 206 (68%) patients received adjuvant chemoradiation, 71 (23%) received adjuvant radiotherapy, and 26 (9%) did not warrant any adjuvant therapy. Relevant patient demographic and clinicopathological data are summarized in **Table 2**. The pathological nodal staging was done using the AJCC8 staging system. All the patients underwent neck dissection. The majority of them were N0, 154 (51%); N1, 16 (5%); N2, 53 (18%); and N3, 80 (26%). Positive bony and mucosal margins were seen in 8 (3%) and 7 (2%) of cases, respectively.

**Table 2:** Demographic, histopathological, and clinical details of the whole cohort (n = 303).

Variable	Bone erosion		p value
	No Bone Erosion/Deep Cortical Erosion	Marrow with or without Mandibular Canal Involvement	
**Gender**			
Male	137	121	0.63
Female	22	23	
**Preoperative treatment**			
None	101	107	
Neoadjuvant chemotherapy (NACT)	56	36	0.19
Radiotherapy (RT)	1	0	
Concurrent chemoradiotherapy (CTRT)	1	1	
**Type of surgery**			
Marginal mandibulectomy	40	2	<0.001
Segmental mandibulectomy	119	142	
**Adjuvant therapy**			
None	23	3	0.001
RT	37	34	
CTRT	99	107	
**Pathological skin involvement**			
Absent	117	119	0.07
Present	42	25	
**Pathological node involvement**			
Absent	86	68	0.25
Present	73	76	
**Extracapsular spread**			
Absent	102	83	0.29
Present	57	61	
**Lymphovascular invasion**			
Absent	156	137	0.2
Present	3	7	
**Perineural Invasion**			
Absent	129	106	0.13
Present	30	38	
**Histopathological grade of tumour**			
Well differentiated	25	13	0.17
Moderate differentiated	106	99	
Poorly differentiated	28	32	
**Mucosal margins**			
Free	157	139	0.26
Positive	2	5	
**Bone margins**			
Free	158	137	0.03
Involved	1	7	
**Pathological T stage AJCC** 8			
T1	17	0	<0.001
T2	34	0	
T3	24	0	
T4a	84	144	
**MMC T staging system**			
T1	23	0	<0.001
T2	50	0	
T3	32	0	
T4a	54	144	
**Pathological Nodal staging**			
N0	86	68	0.07
N1	10	6	
N2	31	22	
N3	32	48	

AJCC8, 8th edition of the American Joint Committee on Cancer; MMC, Marrow and mandibular canal staging system.

### Patterns of Bone Involvement

According to the radiology review, mandibular bone was involved in 187 (62%) patients. Out of these, deep cortical erosion was seen in 43 (23%), marrow was involved in 64 (34%), and mandibular canal involvement was seen in 80 (43%) patients.

### Survival Analysis

**Figure 3:**
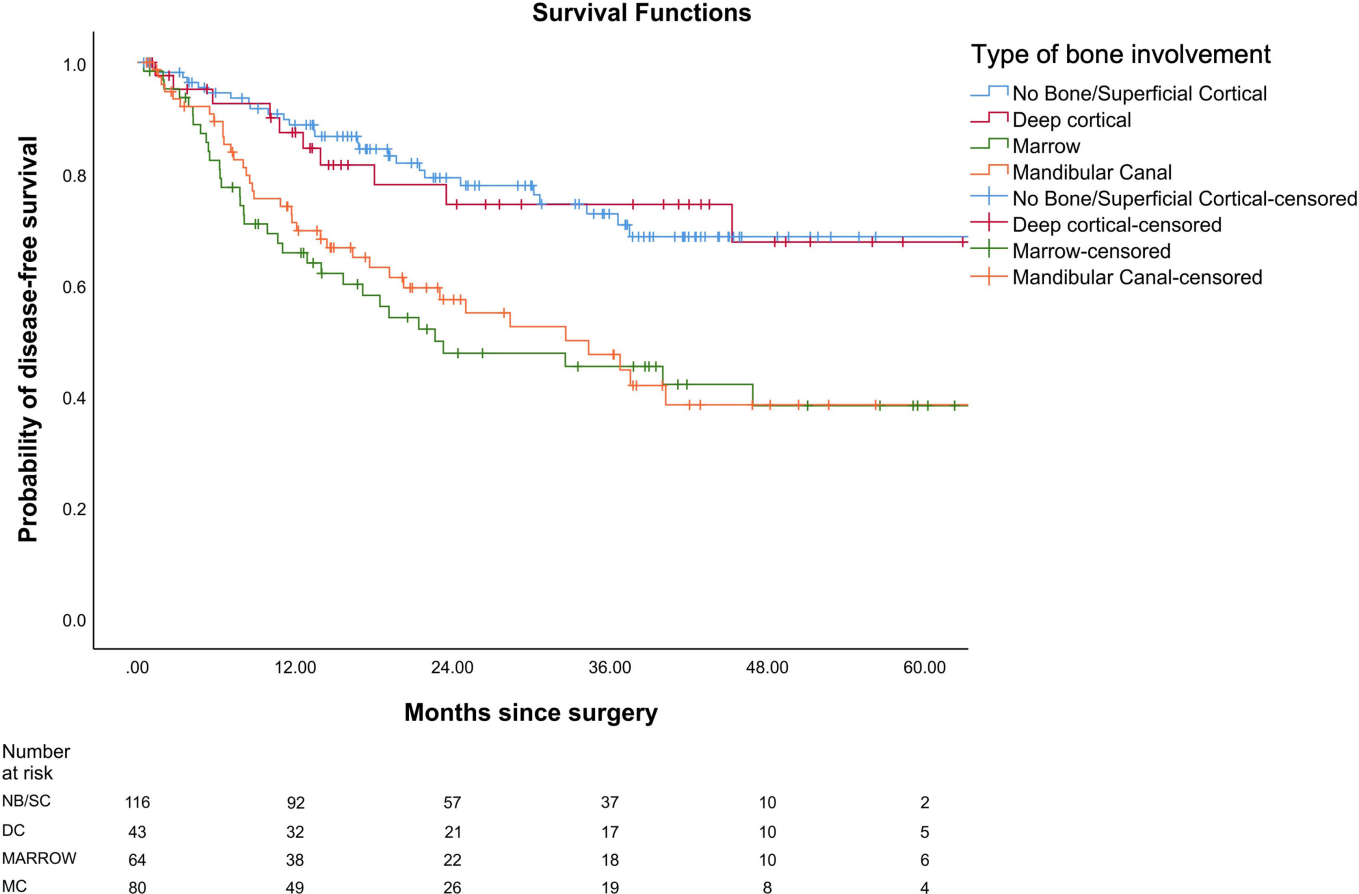
Comparison of Kaplan–Meier survival curves of disease-free survival (DFS) for different patterns of bone involvement.

**Figure 4:**
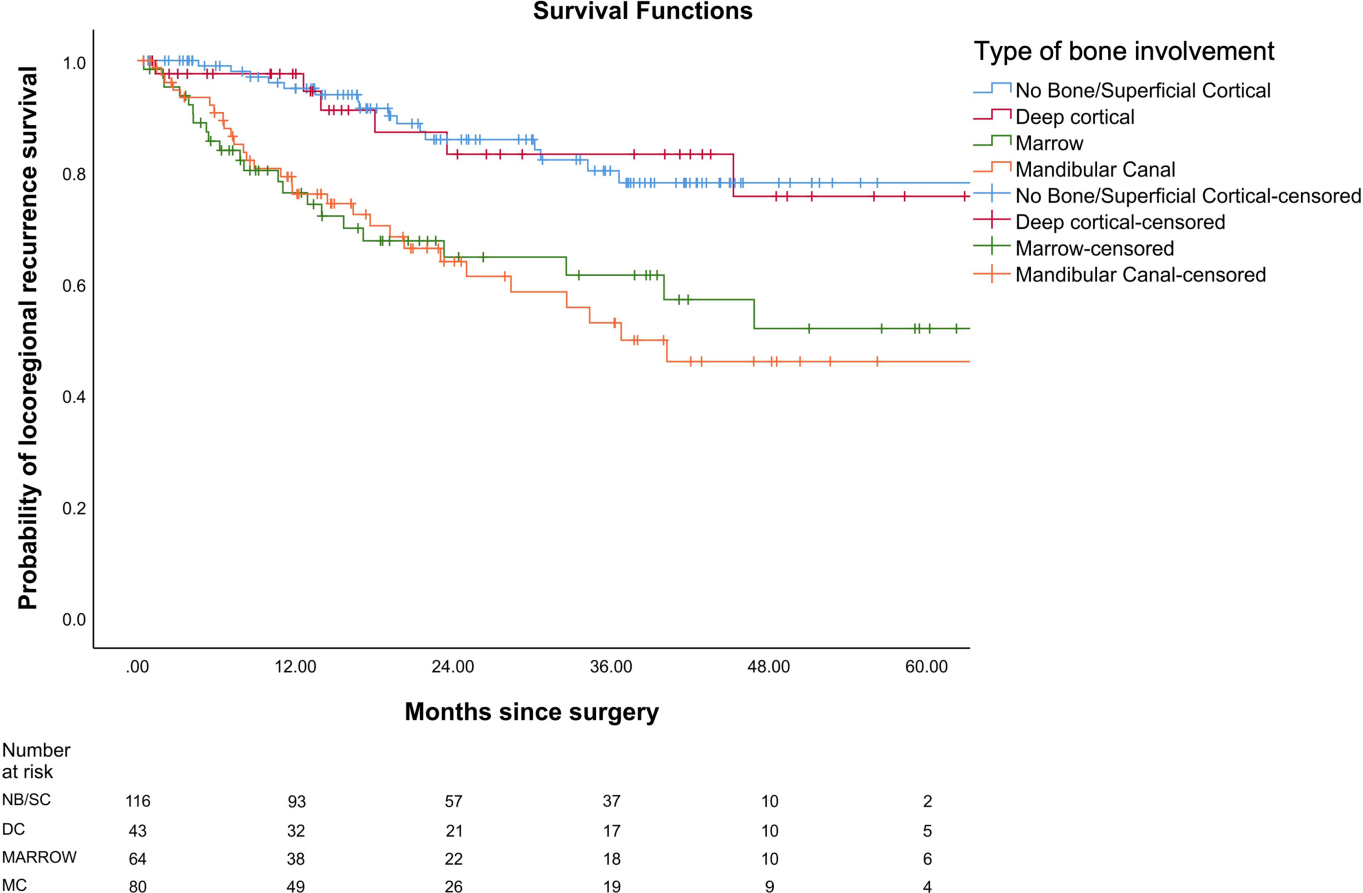
Comparison of Kaplan–Meier survival curves of locoregional recurrence-free survival (LRRFS) for different patterns of bone involvement.

**Figure 5:**
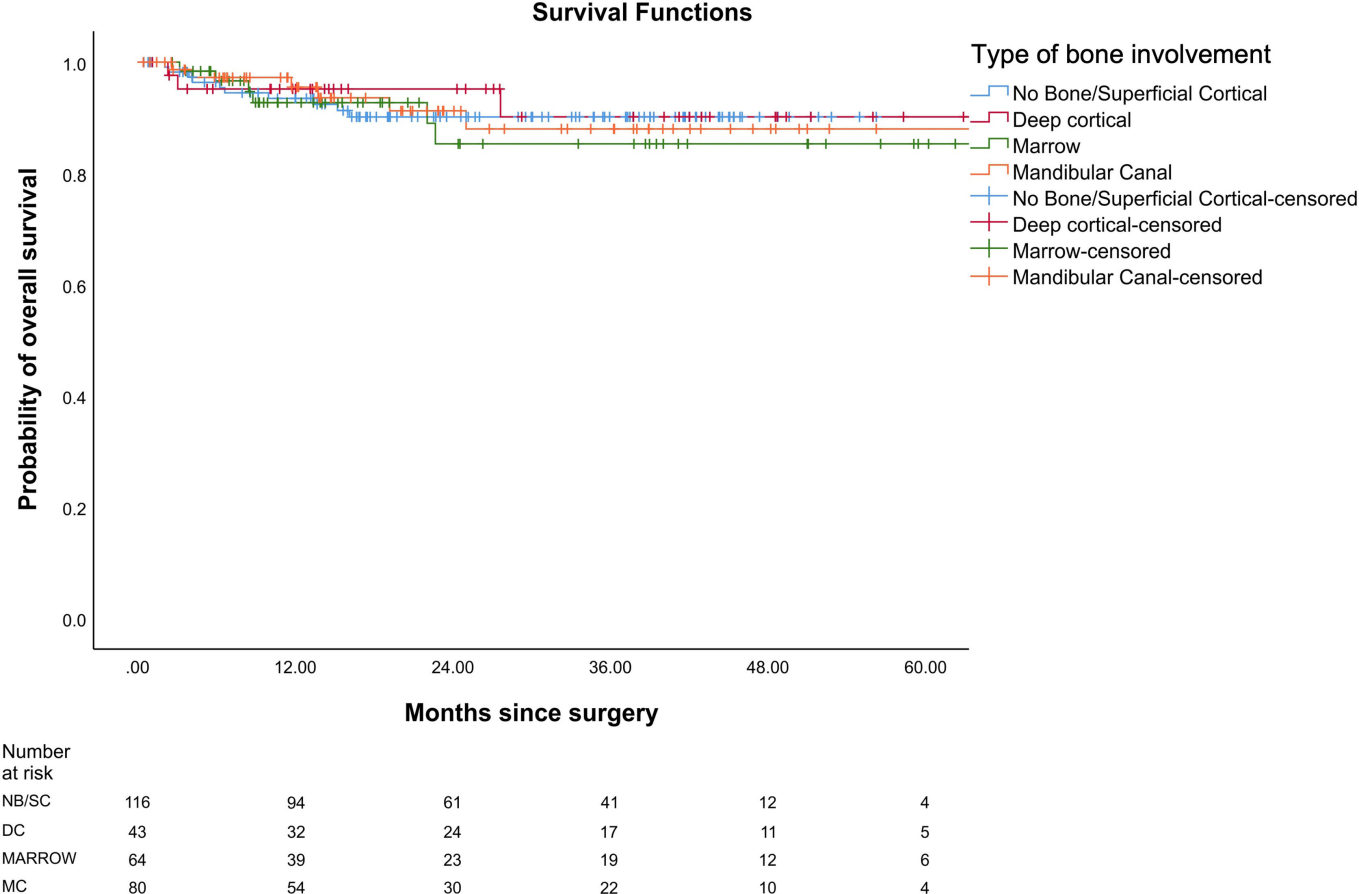
Comparison of Kaplan–Meier survival curves of overall survival (OS) for different patterns of bone involvement.

In our study, the mean OS was 26 months, the mean DFS was 24.6 months, and the mean LRRFS was 24.7 months. The cohorts were stratified based on the type of bone erosion. **Figures 3**–**5** depict the Kaplan–Meier survival curves for the various patterns of bone involvement. No bone erosion or deep cortical bone involvement had a significantly better outcome compared with marrow or mandibular canal involvement (DFS, p = 0.023; LRRFS p = 0.013). However, the difference in OS between the 2 groups was not significant (p = 0.82). Marrow involvement had a similar survival (DFS and LRRFS) to mandibular canal invasion (for DFS, p = 0.59; for LRRFS p = 0.77). There was a significant survival difference (DFS and LRRFS) between the deep cortex and marrow (for DFS, p = 0.005; for LRRFS, p = 0.017) and the deep cortex and mandibular canal involvement (for DFS, p = 0.01; for LRRFS p = 0.006).

When the patients were stratified based on extracapsular spread (ECS), there was statistically worse DFS and LRRFS in patients with marrow/canal involvement as compared with no bone erosion/deep cortical erosion in the ECS-negative subgroup (p = 0.023 and p = 0.013, respectively). However, the difference in the 2 groups was not statistically significant (p = 0.389 for DFS; p = 0.641 for LRRFS) in the ECS-positive subgroup. The type of bone was an independent prognostic factor for DFS on multivariate analysis after making adjustments for known histopathological prognostic factors and retroantral fat involvement involvement p < 0.001(**Table 3**). Other independent prognostic factors were retroantral fat involvement, skin involvement, and tumor grade. The type of bone involvement was the only independent prognostic factor for LRRFS on multivariate analysis, p < 0.001 (**Table 4**).

**Table 3:** Multivariate analysis of disease-free survival in patients with gingivo-buccal squamous cell carcinoma.

Variable	Events (n)	Hazard Ratio	95% CI	p-Value
Bone involvement				
No bone involvement/deep bone involvement	37	0.42	028–0.65	**<0.001**
Marrow with or without mandibular canal involvement	70	1.0	Reference	
Retroantral fat involvement				
Present	8	5.3	2.29–12.26	**<0.001**
Absent	99	1.0	Reference	
Pathological skin Involvement				
Present	28	1.68	1.06–2.65	**0.026**
Absent	79	1.0	Reference	
Pathological node Involvement				
Present	60	1.56	0.81–3.0	0.18
Absent	47	1.0	Reference	
Pathological extracapsular spread				
Present	48	0.81	0.42–1.59	0.56
Absent	59	1.0	Reference	
Lymphovascular invasion				
Present	6	1.31	0.54–3.14	0.54
Absent	101	1.0	Reference	
Perineural invasion				
Present	32	1.04	0.65–1.66	0.86
Absent	75	1.0	Reference	
Histopathological grade of tumor				
Poor	33	2.13	1.36–3.34	**<0.001**
Moderate	67	1.0	Reference	
Histopathological grade of tumour				
Well	7	0.70	0.31-1.57	0.40
Moderate	67	1.0	Reference	
Mucosal Margin				
Positive	2	0.82	0.18-3.62	0.80
Free	105	1.0	Reference	
Bony Margin				
Positive	3	0.52	0.14-1.88	0.32
Free	104	1.0	Reference	

Bold means p value significant.

**Table 4:** Multivariate analysis of locoregional recurrence-free survival in patients with gingivo-buccal squamous cell carcinoma.

Variable	Events (n)	Hazard Ratio	95% CI	p-Value
Bone involvement				
No bone involvement/deep bone involvement	23	0.33	0.19–0.55	**<0.001**
Marrow with or without mandibular canal involvement	51	1	Reference	
Retroantral fat involvement				
Present	4	1.5	0.50–4.49	0.47
Absent	70	1	Reference	
Pathological skin Involvement				
Present	19	1.64	0.94–2.84	0.08
Absent	55	1	Reference	
Pathological node Involvement				
Present	38	0.77	0.31–1.87	0.57
Absent	36	1	Reference	
Pathological extracapsular spread				
Present	32	1.5	0.61–3.71	0.37
Absent	42	1	Reference	
Lymphovascular invasion				
Present	1	0.21	0.02–1.63	0.14
Absent	73	1	Reference	
Perineural invasion				
Present	18	0.89	0.49–1.60	0.71
Absent	56	1	Reference	
Histopathological grade of tumor				
Poor	20	1.55	0.89–2.70	0.12
Moderate	48	1	Reference	
Histopathological grade of tumour				
Well	6	0.7	0.29–1.69	0.44
Moderate	48	1	Reference	
Mucosal Margin				
Positive	2	0.85	0.17.4.21	0.85
Free	72	1	Reference	
Bony Margin				
Positive	3	1.37	0.35–5.26	0.64
Free	71	1	Reference	

Bold means p value significant.

### Marrow and Mandibular Canal Classification—Stage Migration and Comparison With 8th Edition of the American Joint Committee on Cancer

As per the final histopathology report, patients were staged according to the AJCC8 and MMC classifications (**Table 2**). In the MMC classification, patients with deep cortex involvement were downstaged from T4 to the stage according to the size of the tumor and depth of invasion. Out of 228 T4 patients (according to the AJCC8), 30 patients were downstaged to T1–T3. Out of these 30 patients, 6 were restaged to T1, 16 were restaged to T2, and 8 were restaged as T3. When the two staging systems were compared using AIC, the MMC system turned out to be a better staging system for the prediction of survival, as the AIC values of the MMC staging system for LRRFS, DFS, and OS were lower compared with those of the AJCC8 (**Table 5**).

**Table 5:** AIC values of AJCC8 and MMC staging system with respect to survival.

Survival	AIC Values	
	AJCC8 Staging	MMC Staging
LRRFS	266.61	8.92
DFS	1,165.56	82.46
OS	818.98	74.26

LRRFS, locoregional recurrence-free survival; DFS, disease-free survival; OS, overall survival; AIC, Akaike information criterion; AJCC8, 8th edition of the American Joint Committee on Cancer; MMC, Marrow and mandibular canal staging system.

### Patterns of Recurrence

**Table 6** shows the pattern of recurrence with respect to the types of bone involvement. Further, we evaluated if the type of bone erosion had any impact on the pattern of recurrence. The recurrence occurred in 23.3% of patients with deep cortical and no bone involvement versus 48.6% of patients with marrow and mandibular canal involvement, which was statistically significant (p = 0.023). There was a statistically significant association between the type of bone erosion and the type of recurrence (p = 0.013).

**Table 6:** Pattern of bone involvement with pattern of recurrence.

Type of Bone Involvement	Number of Recurrences	Site of Recurrence			
		Local	Regional	Distant	Combination
No bone involved and deep cortical bone erosion (159 cases)	37 (23.3%)	17 (46%)	5 (13.5%)	11 (29.7%)	4 (10.8%)
Marrow with or without mandibular canal (144 cases)	70 (48.6%)	43 (61.4%)	8 (11.4%)	14 (20%)	5 (7.1%)

## Discussion

The prognosis of oral squamous cell carcinoma depends upon a multitude of factors. Several of these are included in the staging system. Bone invasion has been considered as an adverse prognostic factor for a long time, thus meriting adjuvant therapy (2). Over the last few decades, it has been shown that superficial cortical erosion for alveolar primaries does not portend a poorer prognosis; such tumors are, therefore, staged according to their size (13).

There have been several studies that have further tried to understand and characterize the type of bone erosion and its effect on prognosis (20, 21). They have differentiated bone erosion as erosive (superficial cortical erosion) and infiltrative (deep cortical, marrow involvement, and mandibular canal involvement) and looked at their impact on the prognosis and survival. It has been observed that cortical bone erosion may not impact survival and only marrow invasion impacts prognosis. A recent meta-analysis found that only marrow invasion impacted overall and disease-free survival (22). On the contrary, few other studies did not show such an association between any type of bone erosion and survival (14–16). Probably this is the reason why staging systems, rather than characterizing the type of bone erosion, continue to mention merely mandibular bone erosion as present or absent.

Studies on this aspect have looked at all subsites of the oral cavity combined. It is prudent to understand that a buccal mucosa or a lower alveolus cancer is more likely to erode mandibular bone as compared with a tongue cancer (8, 9). They cannot be kept on the same pedestal while making any meaningful conclusions regarding upstaging the disease in presence of bone erosion. Another important aspect that these studies have not considered is the pathological depth of invasion, which plays an important role in assessing the prognosis and has recently been incorporated in the AJCC staging system (23, 24). In our study, we utilized the AJCC8 to stage the patients; thus, the depth of invasion was included in the staging process. As mentioned earlier, we only included buccal mucosa and lower alveolus cancer patients in the study, which is the most relevant cohort. We also excluded patients with high infratemporal fossa and maxillary erosion. This was done to exclusively analyze the prognostic impact of the type of mandibular bone erosion on survival.

On multivariate analysis, type of bone erosion had an independent prognostic impact on DFS and LRRFS (p < 0.001 and p < 0.001, respectively) after making adjustments for other prognostic factors (**Tables 3** and **4**, respectively). Deep cortical erosion had survival similar to cases with no bone erosion. In contrast, marrow and mandibular canal involvement had similar survival (DFS and LRRFS), which was statistically worse than that seen with deep cortical erosion and no bone erosion for DFS and LRRFS (**Figures 3**, **4**).

Based on the results of univariate analysis, patients with deep cortical or no bone involvement were included together, and patients with marrow and mandibular canal were included together for further analysis. We found marrow and mandibular canal involvement to be statistically significantly poorer than no bone or deep cortical erosion for DFS and LRRFS (p =0.023 and p =0.013, respectively). It has also been hypothesized by a few studies that mandibular canal involvement may be associated with higher chances of distant metastasis (25–27). In our study, we found a statistically significant association between type of recurrence and the type of bone erosion (p = 0.013).

There have been few studies that have tried to restage the disease based upon the type of bone erosion. As per Ebrahimi et al., cortex involvement had a similar outcome as no bone involvement (13). They proposed a staging system where the disease was upstaged by 1 T category in the presence of marrow invasion. Another study proposed the JSOT classification, where the tumor was classified as T4a only when there was the involvement of the mandibular canal (10, 11). Involvement of the mandibular marrow without canal involvement was classified according to size; however, these patients performed equally badly as those with canal involvement. Bone erosion was completely ignored in another staging system, where the classification was based upon the soft tissue involvement alone (28). They did not consider bone involvement important for staging the tumor. In all these studies, cases without bone erosion were staged as per the size of the tumor. For staging, they had used the 7th edition of the AJCC, where the impact of depth of invasion was not considered. In the present study, we have staged the patients as per the AJCC8 and have considered the depth of invasion for all the patients.

As per the AJCC8 classification system, the tumor is classified as T4a even on mandibular cortical involvement. But the results of our study show that the cortical involvement did not affect the survival of the patient. Hence, we proposed an MMC classification system in which we downstaged tumors with superficial or deep cortical erosion based solely upon their size and depth of invasion (**Table 1**). Only those having marrow involvement with or without mandibular canal involvement were staged as T4a. This staging was labeled as MMC. The results of our study also show that T classification based upon the MMC staging was a better predictor of OS, DFS, and LRRFS as compared with the AJCC8 (**Table 5**).

The limitation of our study is that it is retrospective. We also did not study the impact of superficial bone erosion on the prognosis. Moreover, information about the pattern of invasion on histopathology for these patients was not available. In spite of these limitations, this study provided a large sample size focusing on the relevant subsite of the oral cancers, the buccal mucosa.

## Conclusion

In this study, we found that for GBC-SCC, bone erosion with marrow as well as mandibular canal involvement, and not cortical erosion, is associated with poorer survival outcomes. The marrow with or without mandibular canal involvement has a higher incidence of recurrence, and there was a statistically significant association between the type of bone involvement and pattern of recurrence. T classification based upon the proposed Mahajan et al. MMC staging system, which downstages deep cortical bone involvement, is a better predictor for survival as compared with the AJCC8.

## Data Availability Statement

The original contributions presented in the study are included in the article/supplementary material. Further inquiries can be directed to the corresponding author.

## Ethics Statement

The studies involving human participants were reviewed and approved by IEC TMC. The ethics committee waived the requirement of written informed consent for participation.

## Author Contributions

Study concept: ND, AbM, and AkM. Study design: ND, AbM, and AkM. Data acquisition: ND, AbM, and AS. Quality control of data algorithms: ND, AbM, AkM, and AB. Statistical analysis: ND, AbM, AkM, and AB. Manuscript preparation: all authors. Manuscript editing: ND, AbM, and AkM. Manuscript reviewing: all authors.

## Conflict of Interest

The authors declare that the research was conducted in the absence of any commercial or financial relationships that could be construed as a potential conflict of interest.

## Publisher’s Note

All claims expressed in this article are solely those of the authors and do not necessarily represent those of their affiliated organizations, or those of the publisher, the editors and the reviewers. Any product that may be evaluated in this article, or claim that may be made by its manufacturer, is not guaranteed or endorsed by the publisher.
